# Deciphering evolutionary dynamics of *WRKY* genes in *Arachis* species

**DOI:** 10.1186/s12864-023-09149-z

**Published:** 2023-01-27

**Authors:** Mingwei Chen, Meiran Li, Longgang Zhao, Hui Song

**Affiliations:** 1 Key Laboratory of National Forestry and Grassland Administration On Grassland Resources and Ecology in the Yellow River Delta, College of Grassland Science, Qingdao Agricultural University, Qingdao, China; 2 Qingdao Key Laboratory of Specialty Plant Germplasm Innovation and Utilization in Saline Soils of Coastal Beach, College of Grassland Science, Qingdao Agricultural University, Qingdao, China; 3 High-Efficiency Agricultural Technology Industry Research Institute of Saline and Alkaline Land of Dongying, Qingdao Agricultural University, Qingdao, China

**Keywords:** *Arachis*, Domestication, Homolog, WRKY

## Abstract

**Background:**

Cultivated peanut (*Arachis hypogaea*), a progeny of the cross between *A. duranensis* and *A. ipaensis*, is an important oil and protein crop from South America. To date, at least six *Arachis* genomes have been sequenced. WRKY transcription factors (TFs) play crucial roles in plant growth, development, and response to abiotic and biotic stresses. WRKY TFs have been identified in *A. duranensis*, *A. ipaensis*, and *A. hypogaea* cv. Tifrunner; however, variations in their number and evolutionary patterns across various *Arachis* spp. remain unclear.

**Results:**

WRKY TFs were identified and compared across different *Arachis* species, including *A. duranensis*, *A. ipaensis*, *A. monticola*, *A. hypogaea* cultivars (cv.) Fuhuasheng, *A. hypogaea* cv. Shitouqi, and *A. hypogaea* cv. Tifrunner. The results showed that the WRKY TFs underwent dynamic equilibrium between diploid and tetraploid peanut species, characterized by the loss of old WRKY TFs and retention of the new ones. Notably, cultivated peanuts inherited more conserved WRKY orthologs from wild tetraploid peanuts than their wild diploid donors. Analysis of the W-box elements and protein–protein interactions revealed that different domestication processes affected WRKY evolution across cultivated peanut varieties. WRKY TFs of *A. hypogaea* cv. Fuhuasheng and Shitouqi exhibited a similar domestication process, while those of cv. Tifrunner of the same species underwent a different domestication process based on protein–protein interaction analysis.

**Conclusions:**

This study provides new insights into the evolution of WRKY TFs in *Arachis* spp.

**Supplementary Information:**

The online version contains supplementary material available at 10.1186/s12864-023-09149-z.

## Background

WRKY transcription factors (TFs) play crucial roles in plant growth, development, and response to abiotic and biotic stresses [1–4]. These abiotic and biotic stresses include drought, salt, extreme temperatures, waterlogging, ultraviolet, and various pathogen and insects [1–3]. The plant developmental stages regulated by WRKY TFs include flowering time, senescence, nutrient utilization, and the development of seeds, pollens, stems, and roots plant [2, 3]. WRKY TFs have a conserved WRKY domain, containing a WRKYGQK motif at the N-terminal end and a zinc finger motif at the C-terminal end [1]. WRKY TFs are classified into three groups; I, II, and III [1]. Group I WRKY TFs contain two domains and a zinc finger (C_2_H_2_) motif [1, 2], while group II and III contain a WRKY domain but have different zinc finger motifs (group II contains C_2_H_2_, whereas group III contains C_2_HC zinc finger motif) [1, 2]. Group II is further classified into five subgroups, IIa-IIe [1, 2], of which subgroups IIa and IIb cluster in one clade, and IId and IIe in a different clade [1, 2, 5].

Several WRKY TFs have been identified in diverse plant species at the genome level [6, 7]. Notably, most studies have mainly focused on the structural variation and evolution of WRKY and the prediction of their biological functions based on RNA-seq and quantitative real-time PCR analyses. WRKY TFs have been identified in at least 12 legumes, including *Arachis duranensis*, *Arachis ipaensis*, *Cajanus cajan*, *Cicer arietinum*, *Glycine max*, *Lotus japonicas*, *Lupinus angustifolius*, *Medicago truncatula*, *Phaseolus vulgaris*, *Trifolium pratense*, *Vigna angularis*, and *Vigna radiate* [6]. The WRKYGQK domain reportedly tends to mutate into WRKYGKK [6]. Duplicated WRKY TFs of the 12 legumes had longer polypeptides than the single WRKY TFs [6]. Synteny analysis revealed that segmental duplication event plays a major role in paralog formation in *G. max*, *A. duranensis*, and *A. ipaensis* [8, 9]. Moreover, accumulating evidence also demonstrated that WRKY paralogs and orthologs mainly underwent purifying selection, suggesting that WRKY homologs have conserved functions [6, 8].

Cultivated peanut (*Arachis hypogaea*) is an important oil and protein crop from South America [10, 11]. It is an allotetraploid plant that resulted from a cross between *A. duranensis* and *A. ipaensis* [11–13]. *A. monticola* is a wild allotetraploid plant known to be the direct progenitor of *A. hypogaea* [14]. To date, genome sequencing of at least six *Arachis* species has been completed, including *A. duranensis*, *A. ipaensis*, *A. monticola*, *A. hypogaea* cv. Fuhuasheng, *A. hypogaea* cv. Shitouqi, and *A. hypogaea* cv. Tifrunner [11, 12, 15–17]. The genomic information of the six *Arachis* species provides crucial data for evolutionary studies at the genome level. In *A. duranensis*, duplicated gene pairs have different responses to drought and nematode stress, and old and young duplicate genes have divergent functions [18, 19]. Old duplicate genes mainly participate in lipid and amino acid metabolism and responses to abiotic stresses, while young duplicate genes are preferentially involved in photosynthesis and biotic stress responses [19]. In *A. duranensis* and *A. ipaensis*, gradual selection and purifying pressure act on the somatic tissue-specific and sex-specific genes [20]. A comparison of the genomic structure between wild and cultivated peanuts revealed that the sub-genomes of cultivated peanuts underwent asymmetric evolution [21]. However, no homoeolog expression bias was observed in vegetative tissues between two sub-genomes of *A. hypogaea* except in reproductive tissues [12, 15, 16, 22]. Whole-genome re-sequencing of 203 cultivated peanut varieties was performed to verify the botanical classification of peanuts, and the results revealed that var. peruviana is possibly the earliest variant from tetraploid progenitors [23]. In addition, seed weight and length-related genes have been identified using genome-wide association analysis, and their functions have been verified in *Arabidopsis* [23].

In addition to genome-level analysis, gene family identification has also been used to study *Arachis* evolution. *A. duranensis*, *A. ipaensis*, and *A. hypogaea* cv. Tifrunner genomes have been used to identify gene families such as nucleotide-binding site-leucine-rich repeat (NBS-LRR), LRR-containing genes, and heat shock transcription factor (HSF) [24–26]. To our knowledge, only the valine-glutamine (VQ) gene family has been compared among the above-mentioned six *Arachis* genomes [27]. The study found that the VQs increased in *A. monticola*, *A. hypogaea* cv. Fuhuasheng, and *A. hypogaea* cv. Shitouqi compared to *A. duranensis* and *A. ipaensis* [27].

Previous studies identified WRKY TFs in *A. duranensis*, *A. ipaensis*, and *A. hypogaea* cv. Tifrunner [8, 28]. However, some coordinates changed, and a few gene models got duplicated in *A. hypogaea* cv. Tifrunner genome [29]. Therefore, this study aimed to identify WRKY TFs in *A. monticola*, *A. hypogaea* cv. Fuhuasheng, *A. hypogaea* cv. Shitouqi, and *A. hypogaea* cv. Tifrunner. We compared the number of WRKY TFs across the various *Arachis* species to determine their homologous relationships and regulatory networks. Therefore, this study provides new insights into the evolution of *Arachis* spp.

## Methods

### Identification of WRKY TFs in *Arachis* species

The released genome sequences of *A. monticola*, *A. hypogaea* cv. Fuhuasheng, *A. hypogaea* cv. Shitouqi, and *A. hypogaea* cv. Tifrunner were obtained from GigaDB (http://gigadb.org/dataset/100453), NCBI (ftp://ftp.ncbi.nlm.nih.gov/genomes/all/GCA/004/170/445/GCA_004170445.1_ASM417044v1), Peanut Genome Resource (http://peanutgr.fafu.edu.cn), and PeanutBase (https://www.peanutbase.org) databases [12, 15–17, 29]. The Hidden Markov Model (HMM) file of WRKY domains (PF03106) was downloaded from the Pfam database [30], and the HMMER program with default parameters was used to identify the WRKY domains among the *Arachis* spp. [31]. The WRKY sequences were extracted using in-house Perl script and were uploaded to the Pfam database to re-confirm the WRKY domains. The identification of WRKY TFs in *A. duranensis* and *A. ipaensis* was based on a previous study [8].

### Phylogenetic tree construction

Six *Arachis* WRKY domains were aligned using MAFFT program [32], and the ProtTest program was used to estimate the best-fit model of maximum likelihood (ML) trees [33]. The ML trees were constructed using the IQ-tree program [34]. The phylogenetic tree visualized using the Figtree program.

### Identification of WRKY paralogs and homoeologs in *Arachis* species

Paralogs occur due to gene duplication events, while homoeologs are formed via polyploidy [35, 36]. In this study, we identified paralogs and homoeologs of the *Arachis* species using the local BLAST program as per the following parameters: (1) the alignment region exceeds 80% of each sequence, (2) sequence identity over 80%, and (3) E-value ≦ 10^–10^ [18, 20, 37, 38].

### W-box *cis*-acting elements of *WRKY* genes in *Arachis* species

WRKY TFs are auto- and cross-regulated by the W-box *cis*-acting elements [27, 39, 40]. In this study, the 2-kb upstream sequences of *WRKY* genes were extracted using the genetic feature format (GFF) by the TBtools program [41]. These sequences were uploaded to the NSITE web service to predict their WRKY binding sites [42].

### Prediction of protein–protein interaction among the WRKY TFs of *Arachis* species

The WRKY TFs were uploaded to the STRING database, and the *A. hypogaea* WRKY sequences were used as a reference for predicting the protein–protein interactions.

## Results

### New WRKY TFs originated from tetraploid *Arachis* species

**Fig. 1:**
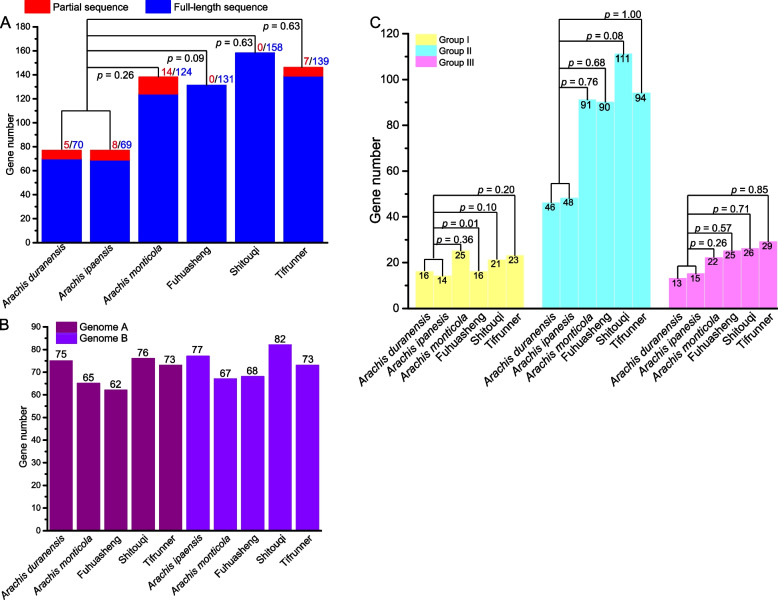
Comparison of *WRKY* genes across various *Arachis* species. **A** Number of *WRKY* genes across various *Arachis* species. **B** Number of *WRKY* genes across various *Arachis* sub-genomes. The excluded *WRKY* genes from *Arachis monticola* and *A. hypogaea* cv. Fuhuasheng due to lack of location information. **C** Number of *WRKY* genes in groups I, II, and III across various *Arachis* species. Statistical analyses were executed using the Chi-square test at *p* ≦ 0.05

The WRKY domains were contained in 138, 131, 158, and 146 sequences of *A. monticola*, *A. hypogaea* cv. Fuhuasheng, *A. hypogaea* cv. Shitouqi, and *A. hypogaea* cv. Tifrunner, respectively (Fig. 1A and Table S1). Among them, 124, 131, 158, and 139 WRKY TFs in *A. monticola*, *A. hypogaea* cv. Fuhuasheng, *A. hypogaea* cv. Shitouqi, and *A. hypogaea* cv. Tifrunner, respectively, were full-length sequences (Fig. 1A). A previous study identified 75 (70 full-length sequences) and 77 (69 full-length sequences) WRKY TFs in *A. duranensis* and *A. ipaensis*, respectively [8]. In this study, the tetraploid and diploid peanut species had an equal number of WRKY TFs (Fig. 1). Similarity, an equal number of WRKY TFs was identified between the sub-genomes and their corresponding ancestral donors (Fig. 1B). The WRKY TFs were classified into three groups: I, II, and III, according to the WRKY domain number and zinc finger type [1, 2]. The number of WRKY TFs in tetraploid peanut species ranged from 16–25, 90–111, and 22–29 in groups I, II, and III, respectively (Fig. 1C). Moreover, the number of WRKY TFs in groups I, II, and III was equal between the tetraploid and diploid peanut species except for group I WRKY in *A. hypogaea* cv. Fuhuasheng (Fig. 1C).

**Fig. 2:**
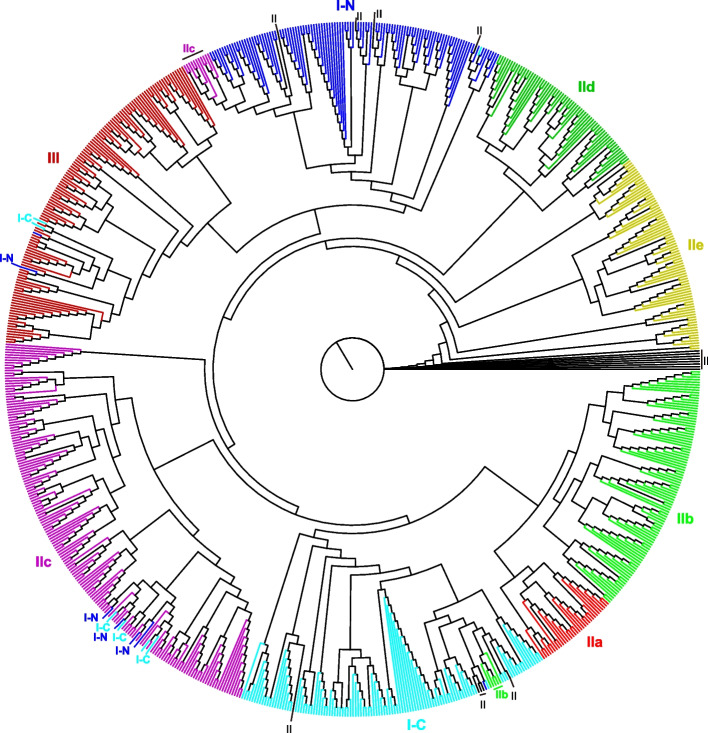
Phylogenetic analysis of *WRKY* genes among various *Arachis* species. The maximum likelihood phylogenetic tree was constructed using the IQ-tree program, and the best-fit model (JTT + I + G) was generated by the ProtTest program. I-N and I-C indicate group I members with WRKY domains from the N- and C-terminal ends

We constructed ML phylogenetic trees using the WRKY domains of *Arachis* spp. and the results showed that WRKY domains were clustered in three major groups: I, II, and III. The group WRKY II domains were further classified into five subgroups: IIa, IIb, IIc, IId, and IIe, consistent with previous studies [1, 2]. However, several group II members from the tetraploid peanut species did not cluster with the corresponding group members from the diploid peanut species (Fig. 2 and Table S1). This indicated that novel WRKY TFs originated from the tetraploid peanut. In addition, several members of subgroups IIb and IIc were clustered with those in subgroups Ic and In, respectively (where In and Ic represent group I members with their WRKY domain on the N-terminal and C-terminal ends (Fig. 2). Several groups Ic and In members also clustered with group IIc and III (Fig. 2). These results indicate that *Arachis* WRKY TFs have multiple origins and that new WRKY TFs originated from the tetraploid peanuts as opposed to diploid peanut.

### Old WRKY TFs were lost in tetraploid *Arachis* species

**Fig. 3:**
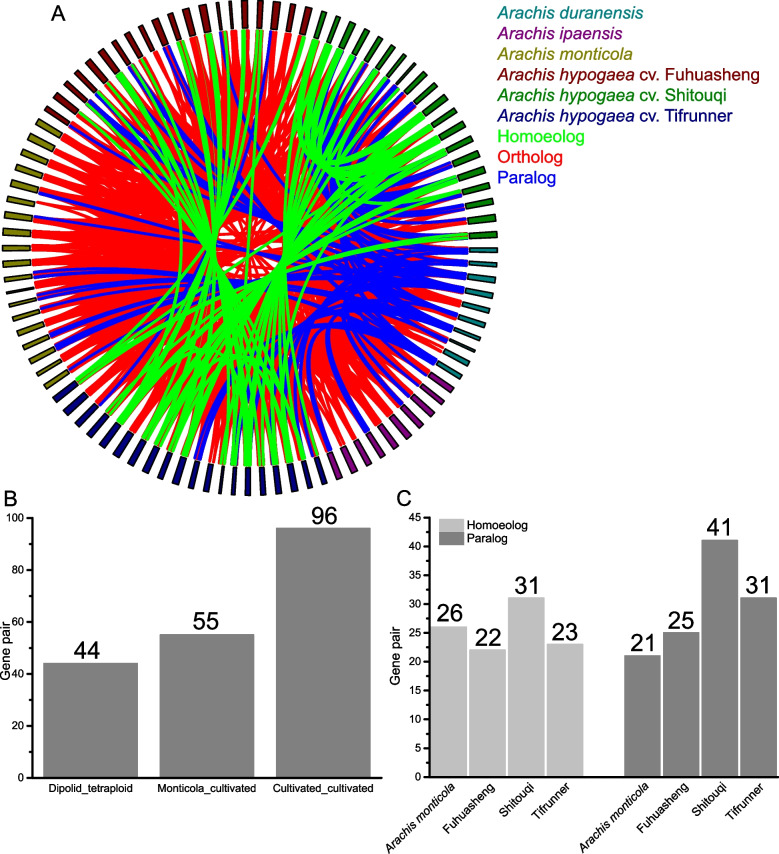
Homologous *WRKY* genes across *Arachis* species. **A** Paralogous, homoeologous, and orthologous *WRKY* genes among various *Arachis* species. **B** Conserved orthologous *WRKY* gene pairs across various *Arachis* species. **C** Paralogous and homoeologous *WRKY* gene pairs across various tetraploid peanut species

Gene expansion and loss occur after a polyploidy event [43], and *A. duranensis* and *A. ipaensis* are the progenitors of tetraploid peanuts [11–13]. Ideally, tetraploid peanut species inherited all the WRKY TFs from wild diploid peanut species; however, only 44 WRKY TFs from two wild diploid peanut species had conserved orthologs with four tetraploid peanut species (Fig. 3A and B). *A. monticola* is known to be the direct ancestor of the cultivated peanut species [14]. In this study, 55 WRKY TFs from *A. monticola* had conserved orthologs in three cultivated peanuts species (Fig. 3A and B). Among the tetraploid peanut species, 96 WRKY orthologous gene pairs were distributed across three cultivated peanuts (Fig. 3A and B). These results indicate that ancestral WRKY TFs were lost after tetraploid formation. Compared with diploid peanut species, cultivated peanuts retained more WRKY TFs from *A. monticola*.

Phenotypic variations such as leaf size, seed size, oil content, flowering pattern, and testa color have been observed across cultivated peanut varieties [10, 44–46]. Notably, *A. hypogaea* cv. Fuhuasheng and *A. hypogaea* cv. Shitouqiare are the breeding parents of about 70% of Chinese peanut cultivars [15, 16]. *A. hypogaea* cv. Tifrunner is a commercial cultivar in America with high disease resistance [47]. Therefore, the three peanut cultivars underwent different evolutionary processes. In this study, orthologs, paralogs, and homoeologs were identified across the tetraploid peanut species. In total, 26, 22, 31, and 23 WRKY homoeologous gene pairs were identified in *A. monticola*, *A. hypogaea* cv. Fuhuasheng, *A. hypogaea* cv. Shitouqi, and *A. hypogaea* cv. Tifrunner, respectively (Fig. 3A and C). Meanwhile, 21, 25, 41, and 31 WRKY paralogous gene pairs were identified in *A. monticola*, *A. hypogaea* cv. Fuhuasheng, *A. hypogaea* cv. Shitouqi, and *A. hypogaea* cv. Tifrunner, respectively (Fig. 3A and C). Compared with *A. monticola*, *A. hypogaea* cv. Fuhuasheng and *A. hypogaea* cv. Tifrunner lost WRKY homoeologs but gained WRKY paralogs, while *A. hypogaea* cv. Shitouqi gained WRKY homoeologs and paralogs (Fig. 3C).

Additionally, 46 WRKY orthologous gene pairs were identified between *A. duranensis* and *A. ipaensis*. Homoeologous WRKY TFs were lost in the tetraploid peanut species compared to the two wild diploid peanut species; however, paralogous WRKY TFs were produced and retained in tetraploid peanut species. Although there was no difference in the number of WRKY TFs between the tetraploid peanut species and their diploid donors, the tetraploid peanut species lost and retained some WRKY TFs, indicating a dynamic equilibrium of WRKY TFs in tetraploid peanut species.

### Domestication affected WRKY evolution in peanut

**Fig. 4:**
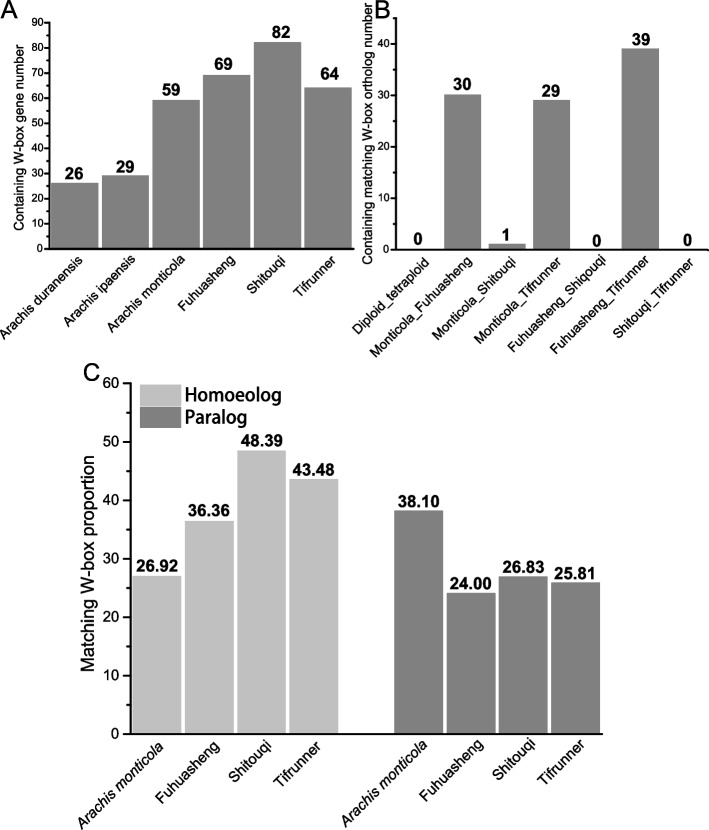
The W-box elements of *WRKY* genes in various *Arachis* species. **A** *WRKY* genes containing W-box elements. **B** The number of orthologous *WRKY* gene pairs containing matching W-box elements. **C** The number of paralogous and homoeologous *WRKY* gene pairs containing matching W-box elements

WRKY TFs exert their biological functions, including auto- and cross-regulation, by binding the W-box elements of *WRKY* genes [3, 7]. In this study, 26, 29, 59, 69, 82, and 64 *WRKY* genes in *A. duranensis*, *A. ipaensis*, *A. monticola*, *A. hypogaea* cv. Fuhuasheng, *A. hypogaea* cv. Shitouqi, and *A. hypogaea* cv. Tifrunner contained at least one W-box element (Fig. 4A). W-box elements of *WRKY* genes were compared among the orthologs, paralogs, and homoeologs of *Arachis* species. The results showed that W-box elements in orthologous *WRKY* genes differed between the diploid and tetraploid peanut species (Fig. 4B). However, matching W-box elements of orthologous *WRKY* genes were found among *A. monticola*, *A. hypogaea* cv. Fuhuasheng, and *A. hypogaea* cv. Tifrunner (Fig. 4B). There were 26.92% (7/26), 36.36% (8/22), 48.39% (15/31), and 43.48% (10/23) homoeologous *WRKY* gene pairs with matching W-box elements in *A. monticola*, *A. hypogaea* cv. Fuhuasheng, *A. hypogaea* cv. Shitouqi, and *A. hypogaea* cv. Tifrunner, respectively (Fig. 4C). This indicated that cultivated peanuts retained more homoeologous W-box elements than wild tetraploid peanuts. In paralogous *WRKY* genes, matching W-box elements were distributed across *A. monticola* (38.10%, 8/21), *A. hypogaea* cv. Fuhuasheng (24.00%, 6/25), *A. hypogaea* cv. Shitouqi (26.83%, 11/41), and *A. hypogaea* cv. Tifrunner (25.81%, 8/31) (Fig. 4C). The results indicated that cultivated peanuts lost more paralogous *WRKY* genes with similar W-box elements than wild tetraploid peanuts. Overall, these results indicated that domestication possibly affected the loss and retention of W-box elements in peanuts.

**Fig. 5:**
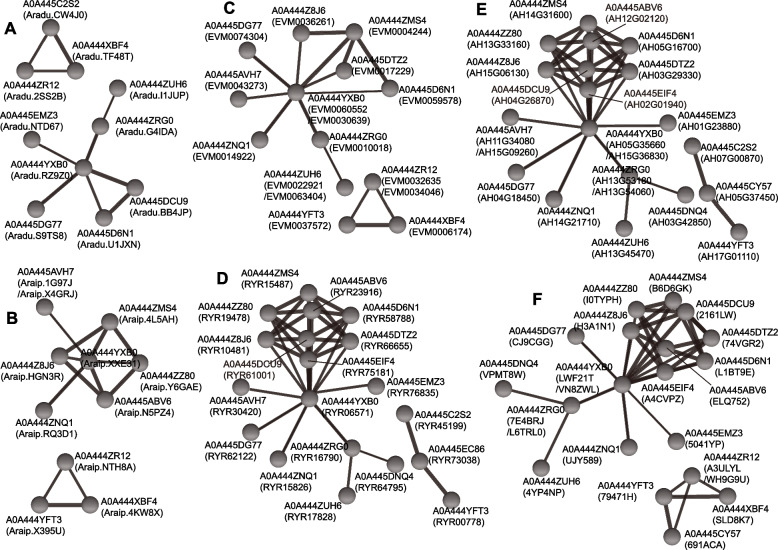
WRKY-WRKY protein interactions across various *Arachis* species. **A** WRKY-WRKY protein interactions in *Arachis duranensis*. **B** WRKY-WRKY protein interactions in *A. ipaensis*. **C** WRKY-WRKY protein interactions in *A. monticola*. **D** WRKY-WRKY protein interactions in *A. hypogaea* cv. Fuhuasheng. **E** WRKY-WRKY protein interactions in *A. hypogaea* cv. Shitouqi. **F** WRKY-WRKY protein interactions in *A. hypogaea* cv. Tifrunner. *A. hypogaea* WRKY sequences were used as a reference for predicting the protein–protein interactions

Studies showed that WRKY-WRKY protein interaction mediates certain biological functions [27, 39]. In this study, protein–protein interactions were assessed using WRKY TFs from diploid and tetraploid peanut species. Compared with WRKY TFs in *A. duranensis* and *A. ipaensis*, more WRKY-WRKY interaction complexes were formed in tetraploid peanut species (Fig. 5). These results indicated that the complex relationships could be due to allopolyploidy. Notably, cultivated peanuts had more complex WRKY protein–protein interactions than wild tetraploid peanuts (*A. monticola*) (Fig. 5), indicating that domestication possibly affects WRKY protein–protein interaction. Furthermore, similar WRKY protein–protein interaction patterns were detected between *A. hypogaea* cv. Fuhuasheng and *A. hypogaea* cv. Shitouqi, while *A. hypogaea* cv. Tifrunner exhibited a distinct WRKY protein–protein interaction relationship.

## Discussion

Several genomes of *Arachis* spp. have been sequenced and publicly released [11, 12, 15–17], accelerating the identification and comparison of gene families at the genome level [8, 28]. However, two factors should be considered when comparing gene families. First, the method used to identify the gene families should be the same because variations in the methodology have been shown to influence the final results. For example, Zhang, et al. [37] found that HMM-based methods are fast and efficient and that using full-length sequences in evolutionary analyses could eliminate false results [37]. Second, various sequencing methods and assembling strategies should be considered when analyzing different *Arachis* genomes, or conserved orthologs among *Arachis* species can be analyzed to avoid variations. A previous study identified 158 WRKY TFs in *A. hypogaea* cv. Tifrunner [28], whose genome was corrected and publicly released in the Peanutbase database [29]. In this study, we identified 146 WRKY TFs from the updated *A. hypogaea* cv. Tifrunner genome. Nevertheless, 158 WRKY TFs identified in the previous study contained 146 WRKY TFs from the updated genome. We utilized the same method to identify and analyze the evolution of WRKYs in *Arachis* spp.

Cultivated peanuts underwent allotetraploidy and domestication [13, 21, 23, 44]. Studies showed that cultivated peanuts have more photosynthetic pigments and larger leaves, stomata, and epidermal cells than their diploid donors because of their allotetraploid genomes [44]. Notably, Leal-Bertioli, et al. [48] compared drought tolerance among *A. duranensis*, *A. ipaensis*, synthetic allotetraploid (*A. duranensis* x *A. ipaensis*)^4x^, and *A. hypogaea* cv. Tifrunner and found that synthetic allotetraploid and *A. hypogaea* cv. Tifrunner had similar but lower drought tolerance than *A. duranensis* and *A. ipaensis* [48]. These findings indicate that the hybrid vigour and not allotetraploidy reduces drought tolerance in tetraploid peanuts more than in their diploid progenitors. Furthermore, a comparison of drought genes between *A. hypogaea* cv. Tifrunner and two diploid donors showed that *A. hypogaea* cv. Tifrunner lost ancestral drought genes, while new copies of drought tolerance genes lack origin function after allotetraploidy [49]. We found that tetraploid peanuts lost the old WRKY TFs and retained the new ones. Based on the changes in drought-tolerance genes of *A. hypogaea* cv. Tifrunner, we hypothesized that new WRKY TFs possibly have new functions and formed complex regulatory networks in tetraploid peanut species.

This study showed that domestication affected *WRKY* genes in cultivated peanuts*.* The number of W-box elements in *WRKY* genes was affected in cultivated peanuts compared with *A. monticola*. In addition, WRKY protein–protein interaction results showed that *A. hypogaea* cv. Fuhuasheng and Shitouqi had similar protein interaction relations, while *A. hypogaea* cv. Tifrunner had a different protein interaction pattern. *A. hypogaea* cv. Fuhuasheng and Shitouqi are the progenitors of the Chinese peanut [15, 16], suggesting that the two *A. hypogaea* cultivars possibly underwent a similar domestication process. *A. hypogaea* cv. Tifrunner was bred in the USA in 2007 and is highly resistant to various diseases, unlike the Chinese peanut [47]. This shows that the differences in the domestication process may be the main reason for the structural and functional variation of the WRKY TFs among the three cultivated *A. hypogaea* cultivars.

## Conclusions

This study identified WRKY TFs in six *Arachis* species. The number of WRKY TFs and their evolutionary patterns were compared, and the results revealed dynamic equilibrium in the number of WRKY TFs across the six *Arachis* spp. Notably, new WRKY TFs were retained while the old ones were lost after allotetraploidy. The present study also showed that domestication affected the WRKY TFs of cultivated peanuts. The WRKY TFs of *A. hypogaea* cv. Fuhuasheng and Shitouqi were subjected to a similar domestication process, while those of cv. Tifrunner underwent a different domestication process based on the protein–protein interaction analysis.

## Supplementary Information

**Additional file 1: Table S1.** WRKY genes in various Arachis species.

## Data Availability

The datasets generated and/or analyzed during the current study are available in the public databases as followings. *Arachis monticola* genome from GigaDB: http://gigadb.org/dataset/100453 *Arachis hypogaea* cv. Fuhuasheng genome from NCBI: ftp://ftp.ncbi.nlm.nih.gov/genomes/all/GCA/004/170/445/GCA_004170445.1_ASM417044v1 *Arachis hypogaea* cv. Tifrunner genome from PeanutBase: https://www.peanutbase.org *Arachis hypogaea* cv. Shitouqi genome from NCBI: accession number is PRJNA480120.

## Abbreviations

GFF: Genetic feature format
HMM: Hidden Markov Model
HSF: Heat shock transcription factor
ML: Maximum likelihood
NBS-LRR: Nucleotide-binding site-leucine-rich repeat
TF: Transcription factor
VQ: Valine-glutamine

## References

[1] Eulgem T, Rushton P, Robatzek S, Somssich I (2000). The WRKY superfamily of plant transcription factors. Trends Plant Sci.

[2] Rushton P, Somssich I, Ringler P, Shen Q (2010). WRKY transcription factors. Trends Plant Sci.

[3] Chen F, Hu Y, Vannozzi A, Wu K, Cai H, Qin Y, Mullis A, Lin Z, Zhang L (2017). The WRKY transcription factor family in model plants and crops. Crit Rev Plant Sci.

[4] Chen X, Li C, Wang H, Guo Z (2019). WRKY transcription factors: evolution, binding, and action. Phytopathol Res.

[5] Zhang Y, Wang L (2005). The WRKY transcription factor superfamily: its origin in eukaryotes and expansion in plants. BMC Evol Biol.

[6] Song H, Sun W, Yang G, Sun J (2018). WRKY transcription factors in legumes. BMC Plant Biol.

[7] Rinerson CI, Rabara RC, Tripathi QJ, Shen PJ, Rushton PJ (2015). The evolution of WRKY transcription factors. BMC Plant Biol.

[8] Song H, Wang P, Lin JY, Zhao C, Bi Y, Wang X (2016). Genome-wide identification and characterization of *WRKY* gene family in peanut. Front Plant Sci.

[9] Yin G, Xu H, Xiao S, Qin Y, Li Y, Yan Y, Hu Y (2013). The large soybean (*Glycine max*) WRKY TF family expanded by segmental duplication events and subsequent divergent selection among subgroups. BMC Plant Biol.

[10] Bertioli DJ, Seijo G, Freitas FO, Valls JFM, Leal-Bertioli SCM, Moretzsohn MC (2011). An overview of peanut and its wild relatives. Plant Genet Resour - Characteriz Utiliz.

[11] Bertioli DJ, Cannon SB, Froenicke L, Huang G, Farmer AD, Cannon EKS, Liu X, Gao D, Clevenger J, Dash S (2016). The genome sequences of *Arachis duranensis* and *Arachis ipaensis*, the diploid ancestors of cultivated peanut. Nat Genet.

[12] Bertioli DJ, Jenkins J, Clevenger J, Dudchenko O, Gao D, Seijo G, Leal-Bertioli SCM, Ren L, Farmer AD, Pandey MK (2019). The genome sequence of segmental allotetraploid peanut *Arachis hypogaea*. Nat Genet.

[13] Bertioli DJ, Abernathy B, Seijo G, Clevenger J, Cannon SB (2020). Evaluating two different models of peanut's origin. Nat Genet.

[14] Hilu KW, Stalker HT (1995). Genetic relationships between peanut and wild species of Arachis sect. Arachis (Fabaceae): Evidence from RAPDs. Plant Syst Evol.

[15] Zhuang W, Chen H, Yang M, Wang J, Pandey MK, Zhang C, Chang WC, Zhang L, Zhang X, Tang R (2019). The genome of cultivated peanut provides insight into legume karyotypes, polyploid evolution and crop domestication. Nat Genet.

[16] Chen X, Lu Q, Liu H, Zhang J, Hong Y, Lan H, Li H, Wang J, Liu H, Li S (2019). Sequencing of cultivated peanut, *Arachis hypogaea*, yields insights into genome evolution and oil improvement. Mol Plant.

[17] Yin D, Ji C, Ma X, Li H, Zhang W, Li S, Liu F, Zhao K, Li F, Li K (2018). Genome of an allotetraploid wild peanut *Arachis monticola*: a de novo assembly. GigaScience.

[18] Song H, Sun J, Yang G (2018). Comparative analysis of selection mode reveals different evolutionary rate and expression pattern in *Arachis duranensis* and *Arachis ipaënsis* duplicated genes. Plant Mol Biol.

[19] Song H, Sun J, Yang G (2019). Old and young duplicate genes reveal different responses to environmental changes in *Arachis duranensis*. Mol Genet Genomics.

[20] Song H, Zhang Q, Tian P, Nan Z (2017). Differential evolutionary patterns and expression levels between sex-specific and somatic tissue-specific genes in peanut. Sci Rep.

[21] Yin D, Ji C, Song Q, Zhang W, Zhang X, Zhao K, Chen C, Wang C, He G, Liang Z (2019). Comparison of *Arachis monticola* with diploid and cultivated tetraploid genomes reveals asymmetric subgenome evolution and improvement of peanut. Adv Sci.

[22] Duan Z, Zhang Y, Zhang T, Chen M, Song H (2022). Proteome evaluation of homolog abundance patterns in *Arachis hypogaea* cv. Tifrunner Plant Methods.

[23] Liu Y, Shao L, Zhou J, Li R, Pandey MK, Han Y, Cui F, Zhang J, Guo F, Chen J (2022). Genomic insights into the genetic signatures of selection and seed trait loci in cultivated peanut. J Adv Res.

[24] Wang P, Song H, Li C, Li P, Li A, Guan H, Hou L, Wang X (2017). Genome-wide dissection of the heat shock transcription factor family genes in *Arachis*. Front Plant Sci.

[25] Song H, Guo Z, Chen T, Sun J, Yang G (2018). Genome-wide identification of LRR-containing sequences and the response of these sequences to nematode infection in *Arachis duranensis*. BMC Plant Biol.

[26] Song H, Guo Z, Hu X, Qian L, Miao F, Zhang X, Chen J (2019). Evolutionary balance between LRR domain loss and young NBS-LRR genes production governs disease resistance in *Arachis hypogaea* cv. Tifrunner BMC Genomics.

[27] Zhang T, Wang Z, Zhang Y, Yang G, Song H. Dissection of valine-glutamine genes and their responses to drought stress in *Arachis**hypogaea* cv. Tifrunner. Funct Integr Genom. 2022;22(4):491-501. 10.1007/s10142-022-00847-7 35366145

[28] Zhao N, He M, Li L, Cui S, Hou M, Wang L, Mu G, Liu L, Yang X (2020). Identification and expression analysis of WRKY gene family under drought stress in peanut (Arachis hypogaea L.). PLoS ONE.

[29] Dash S, Cannon EKS, Kalberer SR, Farmer AD, Cannon SB, Stalker HT, Wilson RF (2016). PeanutBase and other bioinformatic resources for peanut. Peanuts Genetics, Processing, and Utilization.

[30] Finn RD, Mistry J, Schuster-Böckler B, Griffiths-Jones S, Hollich V, Lassmann T, Moxon S, Marshall M, Khanna A, Durbin R (2006). Pfam:clan, web tools and services. Nucleic Acids Res.

[31] Finn RD, Clements J, Eddy SR (2011). HMMER web server: interactive sequence similarity searching. Nucleic Acids Res.

[32] Katoh K, Standley DM (2013). MAFFT multiple sequence alignment software version 7: improvements in performance and usability. Mol Bio Evol.

[33] Darriba D, Taboada GL, Doallo R, Posada D (2011). ProtTest 3: fast selection of best-fit models of protein evolution. Bioinformatics.

[34] Nguyen LT, Schmidt HA, von Haeseler A, Minh BQ (2015). IQ-TREE: a fast and effective stochastic algorithm for estimating maximum-likelihood phylogenies. Mol Bio Evol.

[35] Grover CE, Gallagher JP, Szadkowshi EP, Yoo MJ, Flagel LE, Wendel JF (2012). Homoeolog expression bias and expression level dominance in allopolyploids. New Phytol.

[36] Yoo MJ, Szadkowshi E, Wendel JF (2013). Homoeolog expression bias and expression level dominance in allopolyploid cotton. Heredity.

[37] Zhang Y, Yin D, Song H (2020). Genome-wide identification and characterization of gene families in *Arachis*: methods and strategies. Front Genet.

[38] Song H, Gao H, Liu J, Tian P, Nan Z (2017). Comprehensive analysis of correlations among codon usage bias, gene expression, and substitution rate in *Arachis duranensis* and *Arachis ipaënsis* orthologs. Sci Rep.

[39] Yan L, Liu ZQ, Xu YH, Lu K, Wang XF, Zhang DP (2013). Auto- and cross-repression of three Arabidopsis WRKY transcription factors WRKY18, WRKY40, and WRKY60 negatively involved in ABA signaling. J Plant Growth Regul.

[40] Xu X, Chen C, Fan B, Chen Z (2006). Physical and functional interaction betwenn pathogen-induced Arabidosis WRKY18, WRKY40, and WRKY60 transcription factors. Plant Cell.

[41] Chen C, Chen H, Zhang Y, Thomas HR, Frank MH, He Y, Xia R (2020). TBtools: an integrative toolkit developed for interactive analyses of big biological data. Mol Plant.

[42] Shahmuradov IA, Solovyev VV (2015). Nsite, NsiteH and NsiteM computer tools for studying transcription regulatory elements. Bioinformatics.

[43] Van de Peer Y, Mizrachi E, Marchal K (2017). The evolutionary significance of polyploidy. Nat Rev Genet.

[44] Leal-Bertioli SCM, Moretzsohn MC, Santos SP, Brasileiro ACM, Guimaraes PM, Bertioli DJ, Araujo ACG (2017). Phenotypic effects of allotetraploidization of wild *Arachis* and their implications for peanut domestication. Am J Bot.

[45] Kunta S, Chu Y, Levy Y, Harel A, Abbo S, Ozias-Akins P, Hovav R (2022). Identification of a major locus for flowering pattern sheds light on plant architecture diversification in cultivated peanut. Theor Appl Genet.

[46] Zhang K, Yuan M, Xia H, He L, Ma J, Wang M, Zhao H, Hou L, Zhao S, Li P (2022). BSA-seq and genetic mapping reveals *AhRt2* as a candidate gene responsible for red testa of peanut. Theor Appl Genet.

[47] Holbrook CC, Culbreath AK (2007). Registration of 'Tifrunner' peanut. Journal of Plant Registrations.

[48] Leal-Bertioli SCM, Bertioli DJ, Guimarães PM, Pereira TD, Galhardo I, Silva JP, Brasileiro ACM, Oliveira RS, Silva PÍT, Vadez V (2012). The effect of tetraploidization of wild *Arachis* on leaf morphology and other drought-related traits. Environ Exp Bot.

[49] Zhang Y, Chai M, Zhang X, Yang G, Yao X, Song H (2022). The fate of drought-related gene after polyploidization in *Arachis hypogaea* cv. Tifrunner Physiol Mol Biol Pla.

